# A simple and robust real-time qPCR method for the detection of PIK3CA mutations

**DOI:** 10.1038/s41598-018-22473-9

**Published:** 2018-03-09

**Authors:** Virginia Alvarez-Garcia, Clare Bartos, Ieva Keraite, Urmi Trivedi, Paul M. Brennan, Maïwenn Kersaudy-Kerhoas, Karim Gharbi, Olga Oikonomidou, Nicholas R. Leslie

**Affiliations:** 1 Institute of Biological Chemistry, Biophysics and Bioengineering, Heriot-Watt University, Edinburgh, EH14 4AS UK; 2 Edinburgh Cancer Research Centre, University of Edinburgh, Crewe Road South, Edinburgh, EH4 2XR UK; 3 Edinburgh Cancer Centre, Western General Hospital, Crewe Road South, Edinburgh, EH4 2XU UK; 4 Division of Infection and Pathway Medicine, University of Edinburgh Medical School, The Chancellor’s Building, 49 Little France Crescent, Edinburgh, EH16 4SB UK; 5 Edinburgh Genomics, Ashworth laboratories, The University of Edinburgh, Edinburgh, EH9 3JT UK; 6 Department of Clinical Neurosciences, Western General Hospital, Crewe Road South, Edinburgh, EH4 2XU UK

**Keywords:** PCR-based techniques, Cancer genetics

## Abstract

PIK3CA mutations are seemingly the most common driver mutations in breast cancer with H1047R and E545K being the most common of these, accounting together for around 60% of all PIK3CA mutations and have promising therapeutic implications. Given the low sensitivity and the high cost of current genotyping methods we sought to develop fast, simple and inexpensive assays for PIK3CA H1047R and E545K mutation screening in clinical material. The methods we describe are based on a real-time PCR including a mutation specific primer combined with a non-productive oligonucleotide which inhibits wild-type amplification and a parallel internal control reaction. We demonstrate consistent detection of PIK3CA H1047R mutant DNA in genomic DNA extracted from frozen breast cancer biopsies, FFPE material or cancer cell lines with a detection sensitivity of approximately 5% mutant allele fraction and validate these results using both Sanger sequencing and deep next generation sequencing methods. The detection sensitivity for PIK3CA E545K mutation was approximately 10%. We propose these methods as simple, fast and inexpensive diagnostic tools to determine PIK3CA mutation status.

## Introduction

The Phosphoinositide 3-Kinase (PI3K) signalling pathway is a key regulator of many essential cellular functions including survival, proliferation and metabolism^1^. It is one of the most commonly altered signal transduction pathways in human cancers^2,3^ and its aberrant activation also causes mosaic hyper-proliferative syndromes^4,5^. The activation of PI3K signalling observed in cancer and other pathologies appears to be a consequence of mutations in at least one component of the pathway^6^, including the PI3K catalytic subunit alpha (PIK3CA). The identified PIK3CA mutations are dominated by a small number of recurrent mutations located within two major hotspots: E542K and E545K in the helical domain (encoded within exon 9) and H1047R in the kinase domain (encoded within exon 20). These missense mutations each produce a constitutively active form of the PI3K protein that promotes enhanced downstream signalling contributing to cellular transformation^7,8^.

Somatic PIK3CA mutations are present in approximately 40% of all breast cancer tumours^6,9,10^ with a higher frequency in estrogen receptor (ER) positive subtypes^11^. Despite its potential predictive value, the prognostic and therapeutic implications of genetic alterations in the PI3K pathway still remain unclear^12,13^. PIK3CA mutations appear to be associated overall with a better clinical outcome mainly due to their correlation with ER expression^14^ which is considered a good prognostic feature itself. However, activating mutations in the PIK3CA gene have also been proposed as indicative of a poor outcome for Trastuzumab-based therapies in human epidermal growth factor receptor 2 (HER2) positive breast cancer models^15,16^.

Subsets of patients with different PIK3CA mutations might present distinct outcomes or show differential sensitivity to a specific treatment given the distinct biochemical properties of these mutant enzymes^17^. In the COSMIC (Catalogue of Somatic Mutations in Cancer) database, PIK3CA H1047R and E545K represent the two most commonly identified PIK3CA mutations in human tumours, accounting for 55% and 24% respectively of all PIK3CA mutations detected in breast cancer and 38% and 27% respectively of PIK3CA mutations in all cancers. In laboratory experiments, activating PIK3CA point mutation has been presented as a strong driver of tumour development by inducing multipotency^18^ and cell transformation in the mammary gland and being considered an early event in oncogenesis^19^. Several studies suggest that patients harbouring a PIK3CAmutation benefit from PI3K/AKT/mTOR pathway inhibitors^20,21^. Conversely, PIK3CA mutation has been proposed as a negative predictive biomarker to anti-EGFR therapy in metastatic colorectal cancer patients^22^. Consequently, addressing PIK3CA mutation status in cancer patients may provide a more accurate molecular diagnosis enabling tailored therapeutic approaches.

Mutation status in tumours has been often assessed by Sanger DNA sequencing from tissue samples although this method is often limited by its poor sensitivity (~20%) for low abundance mutations. Next-Generation Sequencing (NGS) technologies have enabled high throughput analysis of tumour DNA samples with greatly improved sensitivity^23^. However, due to the complexity and high cost of NGS techniques, their routine implementation into widespread clinical practice seems some way off, particularly in healthcare settings with more limited resources.

Alternative approaches based on the polymerase chain reaction (PCR) principle such as Digital Droplet PCR (ddPCR) and its variant BEAMing or High Resolution Melting Analysis and Amplification-refractory mutation system (ARMS), among others, have been described for the detection of mutant variants in clinical samples^24–26^. Although some of these methods have already been validated in clinical contexts^27,28^, their greater complexity and expense challenge their widespread uptake in clinical laboratories. Therefore there remains a need for cheaper and simpler screening methods that can efficiently detect actionable genetic variants in solid tumours.

Allele-specific PCR strategies, using primers specific for mutant sequences, have been widely used in the clinical detection of DNA sequence variants^29,30^. A method, termed INTPLEX, was recently described to detect oncogenic KRAS and BRAF mutations in plasma samples^31^. Here we use this approach to detect PIK3CA H1047R and E545K point mutations with a method based on standard Sybr Green real-time PCR. Given the simplicity of the technique, this analysis could be potentially performed in any lab with real-time PCR capability and its principle could be easily adapted to other clinically relevant point mutations.

## Results

### Method design

**Figure 1:**
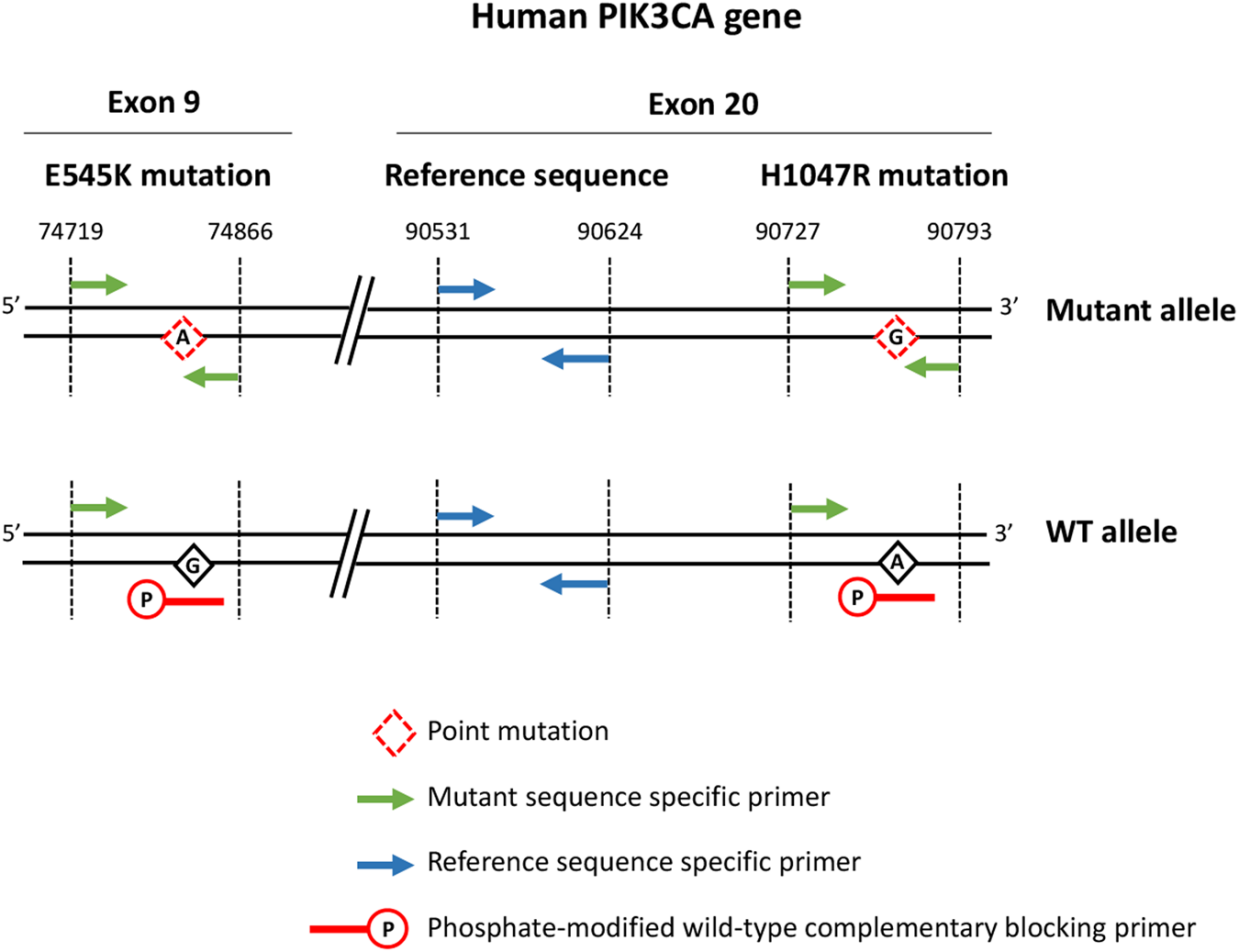
Primer design. The diagram illustrates the position of the primers used for detection of human PIK3CA E545K and H1047R mutations by real-time quantitative PCR. Two separate qPCR reactions are conducted for each mutation detection: a control reaction illustrated with blue arrows representing the primers and a reaction designed to amplify selectively the mutant H1047R or E545K encoding sequences, illustrated with two green arrows (forward and reverse) and an internal red blocking primer. The 3′ end of the reverse orientation oligonucleotide targeting the mutant-specific sequence is anchored to the point mutation site to increase the selectivity of the assay. The reverse orientation blocking primer complementary to a wild-type sequence (red) is phosphorylated at its 3′ end to prevent amplification of the wild-type allele. This blocking oligonucleotide has been designed to partially overlap the mutant-specific primer sequence and to have the variant base approximately in the middle of the primer sequence. The amplicon that serves as a wild-type internal control is located 103 bp upstream of the PIK3CA H1047R mutant amplicon targeting a genomic DNA-based sequence (blue primers).

We sought to develop an assay that could accurately test the PIK3CA H1047R and E545K mutation status in human-derived samples. Our quantitative PCR (qPCR) approach involves the use of a set of primers specifically designed to target the mutant sequence while minimizing the amplification of mismatched products derived from the wild-type allele (Fig. 1 and Table 1). The antisense oligonucleotide targeting the mutant-specific sequence has the variant base located at its 3′ end in order to minimize cross-amplification of the wild-type template. The use of a non-productive phosphate-modified oligonucleotide complementary to the wild-type sequence is intended to supress the amplification of the wild-type allele. It has been designed with the variant base located approximately in the middle of this blocking primer sequence and to partially overlap with the mutant-specific oligonucleotide to ensure high specificity. Each assay also includes the amplification of a non-mutated internal control sequence in a separate reaction.

**Table 1:** Detailed sequences of the primers and size of the amplicons obtained.

Primer name	Primer sequence	Tm (°C)	Direction	Amplicon size (bp)
PIK3CA MUTANT H104R fw	5′-AACTGAGCAAGAGGCTTTGGAG-3′	60.8	sense	71
PIK3CA MUTANT H104R rv	5′-TTGTTGTCCAGCCACCATGAC-3′	62.8	antisense	—
PIK3CA WT BLOCKER (H1047R SITE)	5′-CCAGCCACCATGATGTGCAT-PHO-3′	63.7	antisense	—
PIK3CA WT1 fw	5′-CATTTGCTCCAAACTGACCA-3′	55.2	sense	94
PIK3CA WT1 rv	5′-GATTGGCATGCTGTCGAATA-3′	55.2	antisense	—
PIK3CA WT2 fw	5′-CGACAGCATGCCAATCTCTTC-3′	59.8	sense	112
PIK3CA WT2 rv	5′-CTAAGGCTAGGGTCTTTCG-3′	56.7	antisense	—
PIK3CA MUTANT E545K fw	5′-GGGAAAATGACAAAGAACAGC-3′	55.9	sense	87
PIK3CA MUTANT E545K rv	5′-TCCATAGAAAATCTTTCTCCTGCTT-3′	58.1	antisense	—
PIK3CA WT BLOCKER (E545K SITE)	5′-CTCCTGCTTAGTGATTTCAG-PHO-3′	55.2	antisense	—

The primer pairs WT1 and WT2 were designed to target either a genomic or an intron-spanning internal control sequence within the human PIK3CA gene respectively.

### Allele specificity

**Figure 2:**
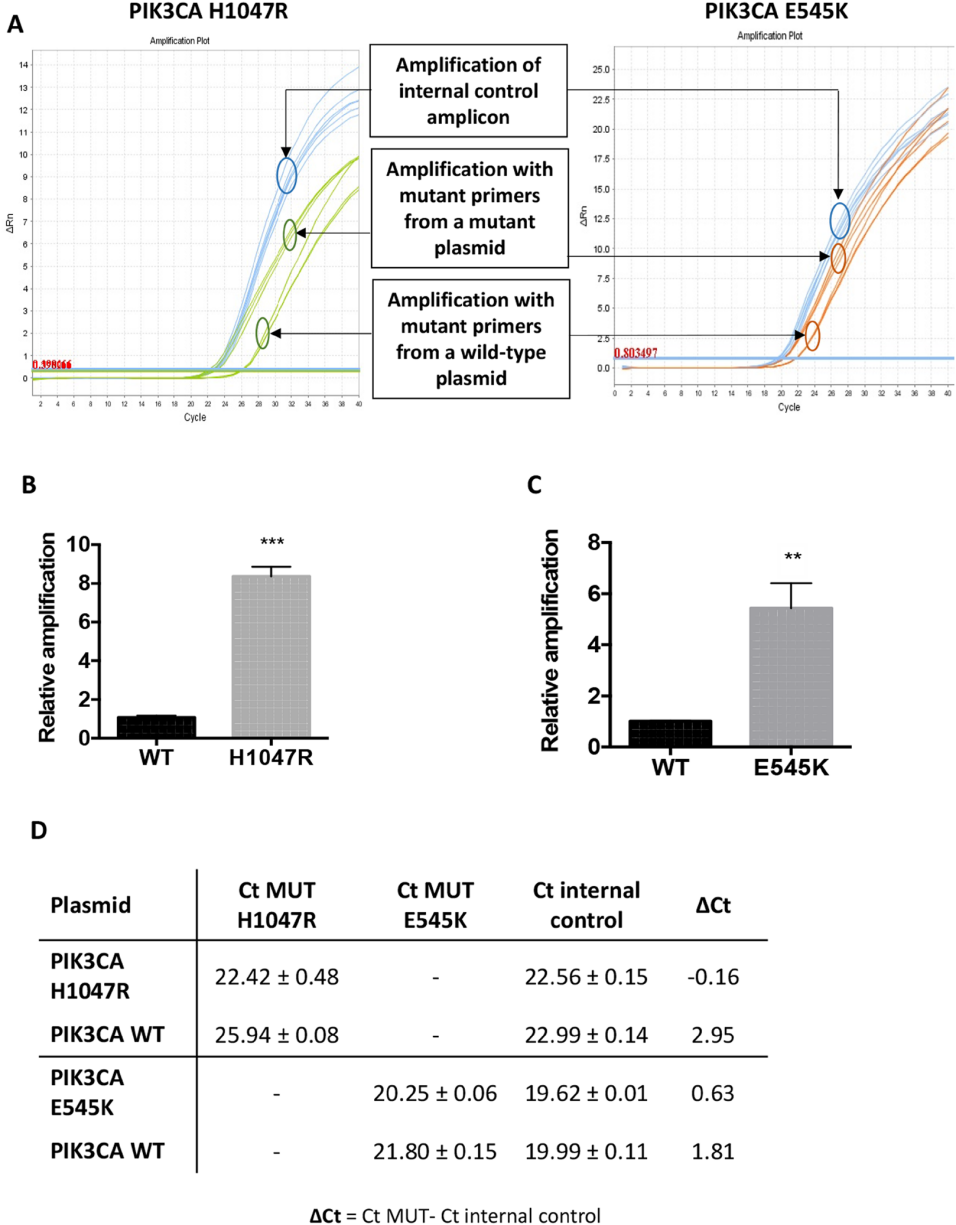
Allele specificity assay. (**A**) Amplification curves obtained from both the mutant-allele specific reaction and the internal control using a total of 1 × 10^6^ copies of plasmid DNA as templates. A plasmid containing the mutant cDNA sequence of the human PIK3CA gene (PIK3CA H1047R or E545K) was used as a standard template for the mutant variant while the plasmid containing the wild-type cDNA sequence of the human PIK3CA gene (PIK3CA WT) served as a negative control. The primer pair WT2 targeting a cDNA sequence within the exon 20 of the PIK3CA gene was used to amplify the internal control amplicon in both mutation detection reactions. qPCR reactions were run in triplicates in three independent experiments (n = 3). (**B**,**C**) Change in fold amplification of the mutant-specific allele of the plasmid PIK3CA H1047R **(B)** or PIK3CA E545K **(C)** harbouring the mutation relative to the amplification of the wild-type PIK3CA WT plasmid. 1 × 10^6^ copies of each plasmid system were subjected to qPCR reactions targeting the mutant-specific sequence and the internal control sequence. qPCR reactions were run in triplicates in three independent experiments (n = 3). Data are expressed as mean ± SEM. ***P < 0.001 compared with PIK3CA WT, **P < 0.01 compared with PIK3CA WT (Student’s *t* test using GraphPad Prism software). (**D**) Ct and ΔCt values of a representative experiment for each mutation detection system showing both the mutant allele and the internal control amplification values of the plasmid standards PIK3CA H1047R, PIK3CA E545K and PIK3CA WT. Data are presented as mean ± standard deviation Ct values for each plasmid sample generated from triplicate assays. ΔCt values were calculated as the difference between the mean Ct value of the mutant allele amplification and the mean Ct value of the internal control amplification.

To evaluate the specificity of the assay we used plasmids containing either the human wild-type PIK3CA cDNA (PIK3CA WT) or the variant cDNA sequence (PIK3CA H1047R or PIK3CA E545K). Each allele-specific qPCR reaction was carried out using 1 × 10^6^ copies of either wild-type or mutant plasmid DNA templates. A shift in the amplification curve of the mutant reaction was observed in the wild-type template compared to the mutant thus indicating the degree of suppression of mismatched amplification **(**Fig. 2A) and suppression was larger when the wild-type blocking primer was included (Figure S1). Although, as expected, both mutant selective qPCR reactions did give a product using wild-type template, the mutant allele amplification reaction showed approximately an 8-fold more efficient product formation from the H1047R mutant template compared to the wild-type plasmid (Fig. 2B) and 5-fold increase in mutant product formation for the E545K reaction (Fig. 2C). The ΔCt values of a representative assay for each mutation detection reactions are provided in Fig. 2D.

### Assay performance in human cell lines

**Figure 3:**
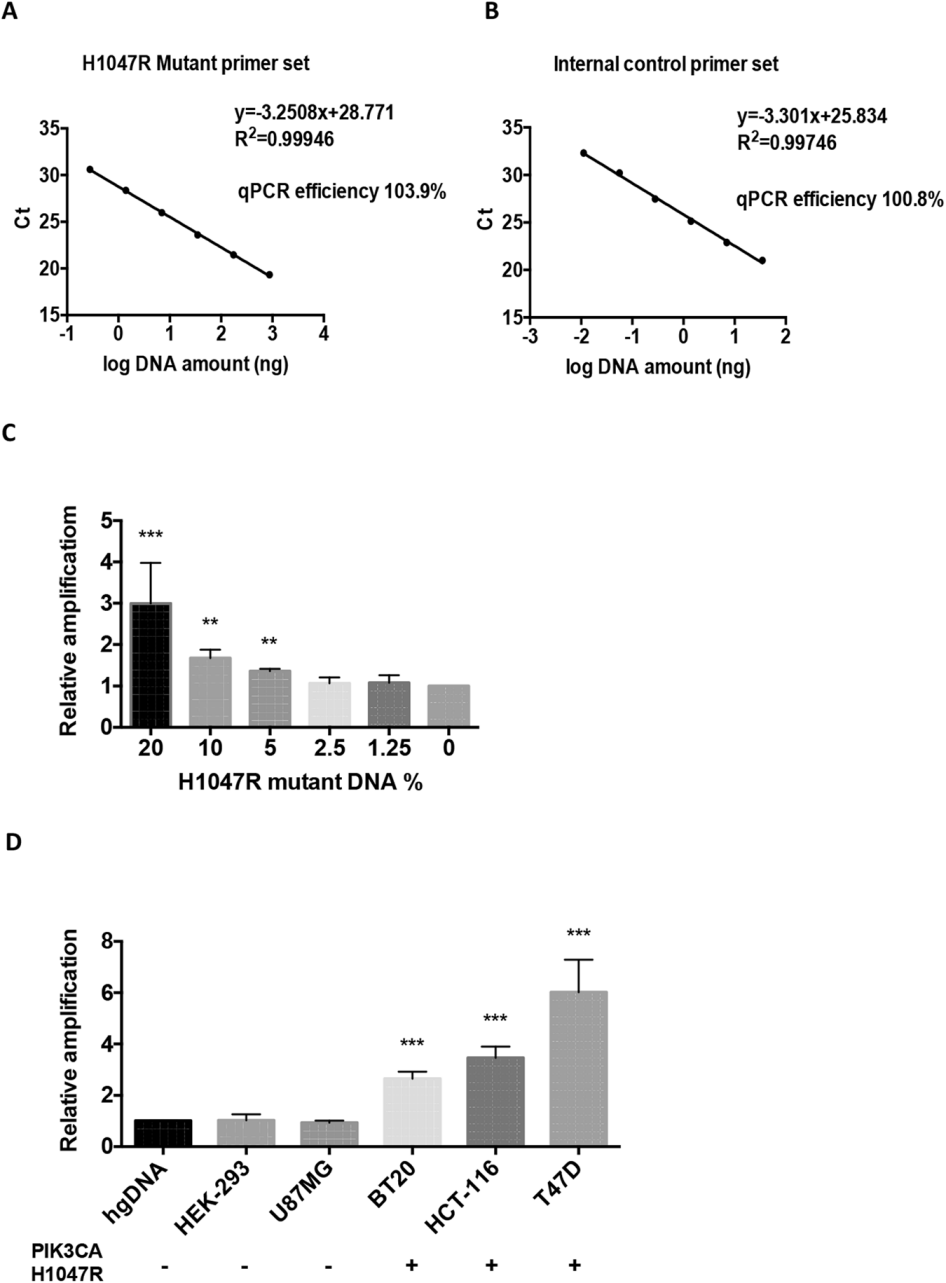
qPCR efficiency, specificity and PIK3CA H1047R mutation detection in cell lines. (**A**,**B**) Genomic DNA from a cell line containing the H1047R mutation was serially diluted 5 times to quantify the efficiency of the mutant-specific (**3A**) and genomic DNA sequence-based internal control (**3B**) primer sets. qPCR efficiency was estimated by using the slope produced by the qPCR standard curve according to the following formula: Efficiency = −1 + 10^(−1/slope)^. Regression curves of a representative experiment for each primer set are shown. All the experimental points were obtained in triplicates. r^2^ values were always ≥0.99. (**C**) Assay sensitivity was assessed in reactions using 5000 template genome copies per reaction. The copy number percentage of a pool of mutant DNA obtained from cell lines containing the mutation (BT20, HCT-116 and T47D cells) was gradually reduced to obtain decreasing ratios of mutant to wild-type DNA. Data are shown as mean Mutant ΔCt values relative to internal control amplification ±SEM. Reactions were run in triplicates and performed 3 times. (n = 3). ***P < 0.001 and **P < 0.01 compared to 0% mutant load (Student’s *t* test using GraphPad prism software). (**D**) Mutation analysis was performed in genomic DNA derived from five cell lines with known PIK3CA H1047R mutation status. Amplification of the mutant allele was significantly increased in the cell lines harbouring the mutation compared to control hgDNA and cell lines with wild-type genotype. Data are shown as mean mutant fold change amplification relative to internal control amplification ±SEM. All the experimental points were obtained in triplicates in three independent experiments (n = 3). ***P < 0.001 compared to hgDNA control (One-way ANOVA test using GraphPad Prism software).

In order to determine the efficiency of each primer set we used genomic DNA extracted from cell lines containing either a PIK3CA H1047R or E545K mutation which was then serially diluted 5 times. As shown in Fig. 3A,B and Figure S2A, the efficiency of the reactions was always close to 100% thus highlighting the suitability of the primer sets and the accuracy of the reactions. The limit of detection in the H1047R reaction was as low as 56 pg while it was approximately 300 pg in the E545K reaction.

To further study the sensitivity of the method in conditions that resemble tumour heterogeneity, we diluted genomic DNA extracted from cell lines containing each mutation into increasing concentrations of wild-type human genomic DNA whilst maintaining a total of 5000 template genomic copies and we then subjected the samples to qPCR analysis. Our results show decreased mutant-allele amplification with decreasing mutant DNA to wild-type ratios (Fig. 3C). The H1047R assay exhibited a detection limit of 5% of diluted mutant content in the sample, when performed in triplicate with a significance threshold of p < 0.05 whereas the E545K assay was less sensitive with a threshold of 10% (Figure S2B) when using a total of 2000 copies per reaction.

We next explored the ability of the method to evaluate the presence of the PIK3CA H1047R mutation in genomic DNA derived from five cell lines with known mutation status. As shown in Fig. 3D, samples harbouring the mutation (genomic DNA obtained from BT20, HCT-116 and T47D cells) displayed a significantly increased relative amplification of the mutant allele compared to a control human genomic DNA sample. However, the samples derived from the cell lines known to have a wild-type genotype (HEK-293 and U87MG) showed amplification values similar to those obtained for control samples. This method could therefore effectively address the mutation status in all cell lines tested. The E545K specific assay was also able to detect this mutation in DNA from MCF-7 and MDA-MB-2361 cells which are recognised to carry this mutation (Figure S2C).

### Analysis of PIK3CA H1047R mutation status in breast cancer biopsy material

**Figure 4:**
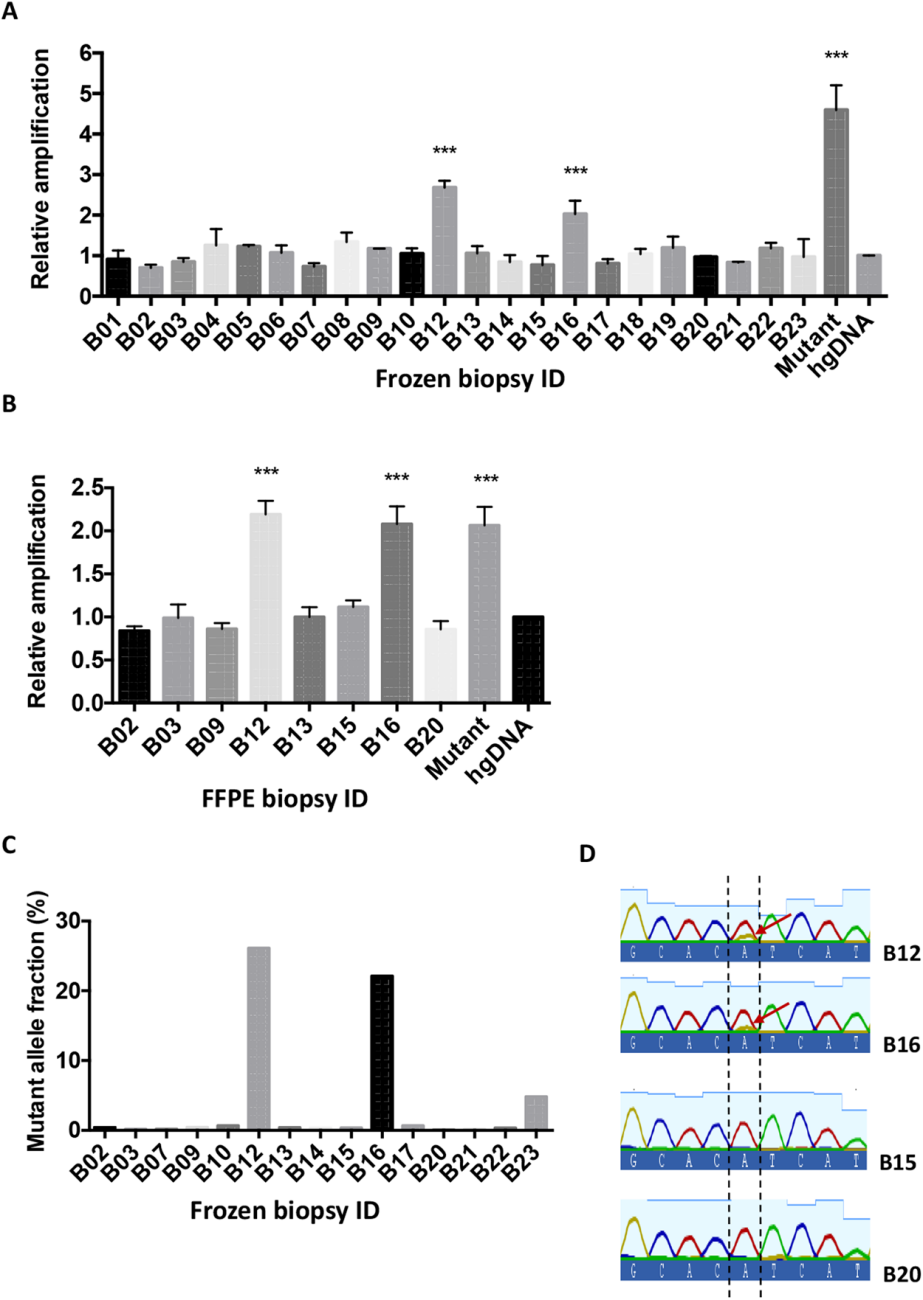
PIK3CA H1047R mutation status analysis in cancer biopsies and FFPE samples. (**A**,**B**) 10 ng of genomic DNA isolated from frozen core biopsies of a total of 22 cancer patients (**A**) or 10 ng of genomic DNA extracted from FFPE sections of 8 patients (**B)** were subjected to qPCR analysis for PIK3CA H1047R mutation. Each assay gave significantly elevated results in the same two samples (patients B12 and B16) in both assays. Data are shown as mean mutant fold change amplification relative to internal control amplification ±SEM. A sample containing a pool of DNA obtained from cell lines carrying the mutation was included in the assay as a positive control. All the experimental points were obtained in triplicates in three independent experiments (n = 3). ***P < 0.001 compared with hgDNA control (One-way ANOVA test). (**C**) Genomic DNA samples isolated from matching frozen core biopsies and white blood cells of 15 breast cancer patients were subjected to targeted deep sequencing using the TruSeq Cancer Amplicon Panel (Illumina). Data are shown as the mutant allele fraction for each sample calculated according to the following formula: G reads/total reads *100, where G reads represent the number of reads obtained for G nucleotide and total reads represent the total number of reads for every nucleotide in genomic position chr3: 178952085, corresponding to the genomic location of the human PIK3CA H1047R variant (GrcH37/hg19 assembly). (**D**) The PCR products derived from the amplification of the PIK3CA sequence encoding exon 20 in four tumor samples (B12, B16, B15, B20) were purified and subjected to Sanger sequencing for genotyping. The arrows indicate the presence of both A and G nucleotide signals at the base corresponding to the PIK3CA H1047R mutation in patients B12 and B16 in contrast to the purely wild-type genotype of patients B15 and B20. Details of the mutation nucleotide sequence are shown in the COSMIC database^35^.

We next wanted to assess the performance of the H1047R assay in DNA samples obtained from frozen core biopsies and Formalin-Fixed Paraffin-Embedded (FFPE) samples from breast cancer patients. The clinical characteristics of the patients are shown in Supplementary Table 1. A total of 22 samples with unknown PIK3CA H1047R status were subjected to the analysis (Fig. 4A). Samples were considered positive for the mutation when the relative amplification of the mutant allele differed significantly from the wild-type human genomic DNA control. A pool of genomic DNA derived from cell lines harbouring the PIK3CA H1047R mutation was used as a positive control in all these assays. The test identified two samples (patients B12 and B16) harbouring the H1047R mutation while the rest of the samples appeared negative (n = 22).

Genomic DNA extraction from FPPE samples typically yields fragmented DNA that is often considered unsuitable for its use in many downstream applications. In order to assess the performance of our assay in FFPE material, genomic DNA was extracted from FFPE blocks from 8 different patients (including B12 and B16) and samples were then subjected to PIK3CA H1047R mutation analysis (Fig. 4B). Mutation status assignments were made based on the relative mutant amplification compared to a control human genomic DNA sample. A positive control containing a pool of DNA derived from 3 cell lines harbouring the mutation was also included in the assay. The analysis again showed a significantly increased relative amplification of the mutant allele in FFPE samples from patients B12 and B16 compared to the non-mutated human genomic DNA control, consistent with the presence of PIK3CA H1047R mutation in these patients. The rest of the samples tested showed a wild-type genotype. To validate the data obtained using our qPCR approach in clinical samples, we were able to subject 15 of these samples to targeted deep sequencing (TruSeq Amplicon Cancer Panel, Illumina) giving a measured allele frequency (AF) for the PIK3CA H1047R mutation in each tumour and white blood cell reference sample (Fig. 4C and Supplementary Table 2). These data confirm the presence of the PIK3CA H1047R A > G substitution in the two patients that were called positive using our PCR method (B12 and B16). Although the mutation was not detected above 5% AF in other samples, sample B23 appeared to carry the mutation very close to this level (AF = 4.8%) and this mutant genotype had not been detected by our qPCR method. Finally, the presence of PIK3CA H1047R mutant DNA was also analysed by PCR amplicon Sanger sequencing in samples B12 and B16, as well as the wild-type genotype of two samples that had been called negative for the mutation (B15 and B20) in our qPCR assay (Fig. 4D). In these data, an evident but small signal corresponding to the H1047R mutation was observed with samples B12 and B16 which was absent with samples B15 and B20.

## Discussion

Here we describe a simple allele-specific quantitative PCR (qPCR) method to detect the common oncogenic PIK3CA H1047R and E545K mutations in human genomic DNA. The assays reported herein rely on the combined use of a set of mutant allele-specific primers with a non-productive phosphate-modified oligonucleotide complementary to the wild-type sequence that blocks the wild-type allele amplification thus achieving high specificity. A separate internal control reaction allows the quantification of the qPCR product.

In reference samples this approach is sensitive enough to detect down to 5% H1047R mutant DNA against a background of 95% wild-type sequences and down to 10% mutant DNA for the E545K mutation. The method successfully identified two PIK3CA H1047R positive patient samples from a test set of available tumour biopsy DNA samples purified either from fresh frozen tumour tissue or from formalin fixed paraffin embedded (FFPE) material. Of a cohort of 22 breast cancer samples, genomic DNA from fresh frozen core biopsies was prepared from all 22 samples and subjected to the qPCR mutation detection assay, 15 of these samples were subjected to amplicon deep sequencing, and 4 of them were additionally genotyped by Sanger sequencing. Furthermore, for 8 of the tumours DNA was extracted from FFPE material and tested using the qPCR assay. The data obtained were coherent and self-consistent. Using the deep sequencing data as a reference, the qPCR assay identified, using DNA from both fresh frozen and FFPE material, two positive tumours (B12 and B16). These two positive samples had observed mutant allele fractions in the Next Generation Sequencing (NGS) data of 26.1% and 22.1% respectively. Notably, the assay did not detect a tumour (B23) in which NGS analysis detected a substantial number of mutant bases: 213 mutant in 4426 total reads giving a mutant allele fraction (AF) of 4.8%, which is below the limit of detection of this method. Overall, this H1047R detection method achieved high sensitivity detecting 67% of mutant samples (100% of samples >5% AF) and gave no false positives (a selectivity of 100%) in a cohort of 22 patients. The data obtained with the qPCR assay were far clearer than that obtained using targeted Sanger sequencing **(**Fig. 4). The PCR assay to detect the PIK3CA E545K mutation had lower sensitivity (>10% mutant AF) and was not validated using clinical samples.

PIK3CA is probably the most commonly mutated gene found in human breast cancers and is also mutated in many other solid cancer types. The increasing number of PI3K/AKT/mTOR inhibitors that are currently under development for breast cancer and other malignancies suggest the potential value of PIK3CA genotyping as a tool for guiding personalized therapies, although its clinical significance still remains incompletely understood^32^. Targeted screening for and detection of these mutations is routinely achieved using several approaches, which either have low sensitivity (targeted Sanger sequencing) or are expensive and/or complex^33,34^. In the latter categories, non-standard PCR detection methods have been used and clinically validated by a few laboratories [32, 33], proprietary detection kits are commercially available, and targeted NGS and digital droplet PCR can give excellent high sensitivity data but are individually costly and also rely on high cost facilities, trained personnel and often have long turnaround times. The ability of our assay to detect PIK3CA H1047R mutations not only in frozen biopsy tissue but also in FFPE tissue specimens is of particular interest, given the value of archived FFPE blocks and the low quality DNA typically obtained from these samples that sometimes limits their use for genomic analysis in downstream applications. We aim that our qPCR based method can be applied at low cost and quick turnaround times in locations lacking expensive genomics facilities. It should also give more sensitive robust data than targeted Sanger sequencing without the need of proprietary reagents and workflows other than those normally used for Sybr Green real-time qPCR. This will improve our knowledge about the prognostic and therapeutic implications of these mutations and further facilitate the development of more individualized PIK3CA targeted therapies.

## Material and Methods

### Study population, recruitment and sample collection

Matching fresh frozen core biopsies, blood samples and formalin-fixed paraffin-embedded (FFPE) samples from 22 female patients with a newly diagnosed breast cancer were collected by the Oncology team of the Western General Hospital in Edinburgh, UK. The study was approved by the Scotland Research Ethics Committee (15/ES/0094). Informed consent was obtained from all individual participants included in the study. All procedures performed in studies involving human participants were in accordance with the ethical standards of the institutional and/or national research committee and with the 1964 Helsinki Declaration and its later amendments or comparable ethical standards. Blood samples were collected in EDTA tubes pre-operatively from patients before receiving any cancer treatment. Blood samples were centrifuged for 10 minutes at 4000 rpm for plasma separation and white blood cell isolation. Tumour tissue, plasma and white blood cells samples were snap frozen and stored at −80 °C until use.

### Plasmids

pBabe puro HA PIK3CA H1047R containing the mutant cDNA sequence of the human PIK3CA gene was from Jean Zhao (Addgene plasmid #12524). PF-Dual-6His-PI3Kalpha/p85 plasmid containing the wild-type cDNA sequence of the human PIK3CA gene and PF-Dual-6His-PI3Kalpha E545K plasmid containing the E545K mutation were kindly provided by James Hastie and Hilary McLaughlin (DU1468 and DU12812 plasmids, respectively, Division of Signal Transduction Therapy, University of Dundee). Plasmids were purified using the QIAfilter Plasmid Maxi kit (Qiagen) and DNA concentration was measured spectrophotometrically (PolarStar Omega, BMG Labtech). Both plasmids were subjected to sequencing for validation.

### Cell lines

The human BT20, HCT-116, T47D, HEK293, MCF-7 and MDA MB-361 and U87MG cancer cells (European Collection of Authenticated Cell Cultures) were maintained in Dulbecco’s Modified Eagle Medium (Life Technologies) containing 10% foetal bovine serum (Invitrogen) and 100 units/ml penicillin-streptomycin (Sigma-Aldrich). Cells were cultured at 37 °C under a 5% CO_2_ atmosphere. BT20, HCT-116, T47D cells were known to possess a heterozygous PIK3CA H1047R mutation while MCF-7 and MDA MB-361 were known to possess a heterozygous PIK3CA E545K mutation. Cell lines HEK293 and U87MG were known to be wild-type for the PIK3CA gene.

### Genomic DNA extraction

Tumour Genomic DNA was extracted from fresh frozen core biopsies (25 mg) retrieved from the tumour rich area using the QIAmp DNA mini kit (Qiagen) according to the manufacturer’s instructions in an elution volume of 100 µl. The extraction of genomic DNA from cell lines in culture was performed using the DNeasy Blood & Tissue kit (Qiagen) according to the manufacturer’s instructions. FFPE blocks were cut in 10 µm sections and genomic DNA was extracted from 4 sections per patient using QIAmp DNA FFPE Tissue kit (Qiagen) according to the manufacturer’s specifications. DNA samples were eluted in 30 µl Tris buffer pH 8. RNase A (Qiagen) was used in all FFPE-derived samples. Genomic DNA was purified from white blood cells after isolation from blood using the QIAamp DNA Blood Mini Kit (Qiagen) according to the manufacturer’s instructions in an elution volume of 200 µl. DNA concentration was determined by spectrophotometry (PolarStar Omega, BMG Labtech). All DNA samples were aliquoted and stored at −80 °C until use.

### PIK3CA H1047R and E545K mutation analysis by real-time quantitative PCR

Allele-specific amplification targeting either the PIK3CA H1047R or PIK3CA E545K mutations in human genomic DNA samples were performed in a Step One Plus real-time PCR system (Applied Biosystems) as follows. Primer3 software version 0.4.0 was used to assist primer design. All primers were purchased from Eurofins Genomics. A list of the oligonucleotide primers used for each assay is provided in Table 1. Reactions to amplify specifically the mutant sequence were carried out in triplicates in a reaction volume of 12.5 µl using 6.25 µl of 2x Power Sybr Green master mix (Life Technologies), 1.25 µl of each amplification primer (final concentrations 100 nM of forward and reverse primers, 200 nM of the blocking primer) and 2.5 µl of purified genomic template DNA (10 ng). Reactions targeting the internal control sequence were performed according to the same protocol using the same primer final concentrations but adding 1.25 µl of nucleic acid-free water (Thermo Fisher Scientific) instead of the blocking primer. Cycling conditions consisted on an initial denaturation step of 95 °C for 10 minutes followed by 40 cycles of 95 °C denaturation for 15 seconds and an annealing/extension step of 1 min at 60 °C. Post amplification melting curve analysis were performed for each reaction product to confirm the absence of non-specific amplification products. Changes in fold amplification were analysed by the comparative ΔΔCt method. Cycle threshold (Ct) values were recorded for each allele-specific and internal control assay and corresponding 2^−ΔΔCt^ values were calculated. Samples were considered to carry the mutation when the relative amplification of the mutant allele was statistically significant with respect to a control sample. All the samples were compared to genomic DNA (Bioline) derived from human placenta as a wild-type control. A pool of DNA derived from 3 different cell lines carrying PIK3CA H1047R mutation (BT20, HCT-116 and T47D) was used as a positive control for all PIK3CA H1047R mutation status analysis.

### Next-Generation Sequencing

The PIK3CA H1047R status of clinical samples was verified by Next-Generation Sequencing analysis using the Illumina Tru-Seq Amplicon Cancer Panel. Genomic DNA extracted from frozen core biopsies was processed at the Edinburgh Genomics facility according to the manufacturer’s instructions and sequenced on an Illumina MiSeq using 250 base paired-end sequencing strategy (version 2 chemistry). Sequence data was trimmed for primer and low quality sequences using cutadapt (version 1.8.3); and aligned to the reference genome (GrcH37/hg19 assembly) using bwa mem (version 0.7.13). Pairs of normal and tumor samples were provided to MuTect pipeline (version 1.1.7) along with dbSNP (hg19) and COSMIC (version 80) variant files. All candidate mutations that were not rejected as somatic variants by MuTect were retained. VCFtool version 0.1.13 was used to derive the allele frequency at each site.

### Statistical analysis

Statistical analysis was carried out using GraphPad Prism 6 (GraphPad Software Inc). Significant differences between groups were assessed using an unpaired parametric two-tailed *t* test assuming equal standard deviations. One-way ANOVA was used in experiments with more than one group to compare to control. Samples were considered statistically significant when *P* value was lower than 0.05.

### Data availability

The datasets generated during and/or analysed during the current study are available from the corresponding author on reasonable request.

## Electronic supplementary material

Supplementary Information
