# Author Correction: A simple and robust real-time qPCR method for the detection of PIK3CA mutations

**DOI:** 10.1038/s41598-021-95597-0

**Published:** 2021-08-06

**Authors:** Virginia Alvarez-Garcia, Clare Bartos, Ieva Keraite, Urmi Trivedi, Paul M. Brennan, Maïwenn Kersaudy-Kerhoas, Karim Gharbi, Olga Oikonomidou, Nicholas R. Leslie

**Affiliations:** 1 Institute of Biological Chemistry, Biophysics and Bioengineering, Heriot-Watt University, Edinburgh, EH14 4AS UK; 2 Edinburgh Cancer Research Centre, University of Edinburgh, Crewe Road South, Edinburgh, EH4 2XR UK; 3 Edinburgh Cancer Centre, Western General Hospital, Crewe Road South, Edinburgh, EH4 2XU UK; 4 Division of Infection and Pathway Medicine, University of Edinburgh Medical School, The Chancellor’s Building, 49 Little France Crescent, Edinburgh, EH16 4SB UK; 5 Edinburgh Genomics, Ashworth Laboratories, The University of Edinburgh, Edinburgh, EH9 3JT UK; 6 Department of Clinical Neurosciences, Western General Hospital, Crewe Road South, Edinburgh, EH4 2XU UK

Correction to: *Scientific Reports* 10.1038/s41598-018-22473-9, published online 09 March 2018

The original version of this Article omitted the Funding section. The Funding section now reads:

“IK was funded by Medical Research Scotland PhD-883-2015.”

The original Article has been corrected.

