# Supplementary material for: A simple and robust real-time qPCR method for the detection of PIK3CA mutations
Source: Sci Rep. 2018 Mar 9;8:4290. doi: 10.1038/s41598-018-22473-9 (PMC5844869; doi:10.1038/s41598-018-22473-9)
Supplement: Supplementary file 1 — Supplementary Information [file 41598_2018_22473_MOESM1_ESM.pdf]

A simple and robust real-time qPCR method for the detection of PIK3CA mutations

Virginia Alvarez-Garcia[1*], Clare Bartos[2,3], Ieva Keraite[1,4], Urmi Trivedi[5], Paul M. Brennan[6], Maïwenn Kersaudy-Kerhoas[1,4], Karim Gharbi[5], Olga Oikonomidou[2,3], Nicholas R. Leslie[1].

[1]Institute of Biological Chemistry, Biophysics and Bioengineering, Heriot-Watt University, Edinburgh EH14 4AS, UK.

[2]Edinburgh Cancer Research Centre, University of Edinburgh, Crewe Road South, Edinburgh EH4 2XR, UK.

[3]Edinburgh Cancer Centre, Western General Hospital, Crewe Road South, Edinburgh EH4 2XU, UK.

[4]Division of Infection and Pathway Medicine, University of Edinburgh Medical School, The Chancellor's Building, 49 Little France Crescent, Edinburgh, EH16 4SB, UK

[5]Edinburgh Genomics, Ashworth laboratories, The University of Edinburgh, Edinburgh EH9 3JT, UK.

[6]Department of Clinical Neurosciences, Western General Hospital, Crewe Road South, Edinburgh EH4 2XU, UK.

*All correspondence should be addressed to:

Virginia Alvarez Garcia, PhD

Institute of Biological Chemistry, Biophysics and Bioengineering

School of Engineering & Physical Sciences

Heriot-Watt University

EH14 4AS Edinburgh

United Kingdom

Email: v.alvarez_garcia@hw.ac.uk

Tel: +44 131 451 4748

Supplementary figure legends

Supplementary Figure 1

A, C. Amplification curves obtained from both the PIK3CA H1047R **(A)** or PIK3CA E545K **(C)** mutant-allele specific reaction and the internal control using a total of $1x10^6$ copies of plasmid PIK3CA wild-type DNA as template. A plasmid containing the wild-type cDNA sequence of the human PIK3CA gene was used as a standard template to show the ability of the blocker oligonucleotide to prevent mismatched wild-type allele amplification. The primer pair WT2 targeting a cDNA sequence within the exon 20 of the PIK3CA gene was used to amplify the internal control amplicon. qPCR reactions were run in triplicates in three independent experiments (n=3).

B, D. Ct and ΔCt values of a representative experiment for each mutation detection system showing both the mutant allele H1047R **(B)** or E545K **(D)** and the internal control amplification values of the plasmid PIK3CA WT. Data are presented as mean ± standard deviation Ct values for each plasmid sample. ΔCt values were calculated as the difference between the mean Ct value of the mutant allele amplification and the mean Ct value of the internal control amplification. All the experimental points were obtained in triplicates.

Supplementary Figure 2. qPCR efficiency, specificity and PIK3CA E545K mutation detection in cell lines

A. Genomic DNA from a cell line containing the E545K mutation was serially diluted 5 times and qPCR efficiency was subsequently estimated by using the slope produced by the qPCR standard curve according to the following formula: Efficiency = $-1+10^{(-1/slope)}$. The graph shows the regression curve of a representative experiment. All the experimental points were obtained in triplicates. r^2 values were always ≥ 0.99.

C. Assay sensitivity of the PIK3CA E545K mutation detection was assessed in reactions using 2000 template genome copies per reaction. The copy number percentage of a mutant DNA stock obtained from MCF-7 cells containing the E545K mutation was gradually reduced to obtain decreasing ratios of mutant to wild-type DNA. Data are shown as mean Mutant ΔCt values relative to internal control amplification ± SEM. Reactions were run in triplicates and performed 4 times. (n=4). **p<0.01 and *p<0.05 compared to 0% mutant load (One-way ANOVA test using GraphPad prism software).

D. Mutation analysis was performed in genomic DNA derived from five cell lines with known PIK3CA E545K mutation status**.** Amplification of the mutant allele was significantly increased in the cell lines harbouring the mutation compared to control hgDNA and cell lines with wild-type genotype. Data are shown as mean mutant fold change amplification relative to internal control amplification ± SEM. All the experimental points were obtained in triplicates in three independent experiments (n=3). ***$p<0.001$ and **$p<0.01$ compared to hgDNA control (One-way ANOVA test using GraphPad Prism software).

Supplementary table 1

Clinical characteristics of the twenty two breast cancer patients recruited for the study. ER and PR are Allred Immunohistochemistry scores (range 0-8) for Estrogen and Progesterone Receptor staining.

Supplementary table 2

Genomic DNA samples isolated from frozen core biopsies of 15 breast cancer patients were subjected to targeted deep sequencing using the TruSeq Cancer Amplicon Panel (Illumina). DNA extracted from white blood cells was used to provide reference genomic data for each patient. The table shows the total number of reads obtained for each sample in position 178952085 of chromosome 3 corresponding to the genomic location of the human PIK3CA H1047R variant (GrcH37/hg19 assembly) and the number of reads for each alternative nucleotide in each sample. WBC = white blood cells, T=tumor, Ref-base= reference base.

Supplementary Figure S1

A

PIK3CA H1047R

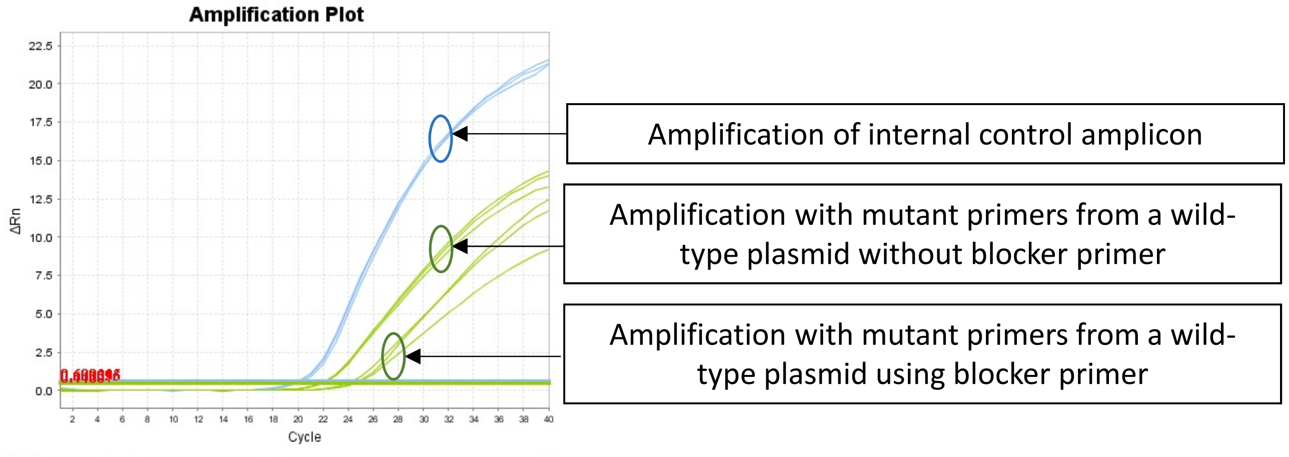

B

WT Plasmid	Ct MUT H1047R	Ct internal control	ΔCt
Without blocker	21.88 ± 0.15	20.11 ± 0.18	1.78
With blocker	24.26 ± 0.33		4.15

ΔCt = Ct MUT H1047R - Ct internal control

C

PIK3CA E545K

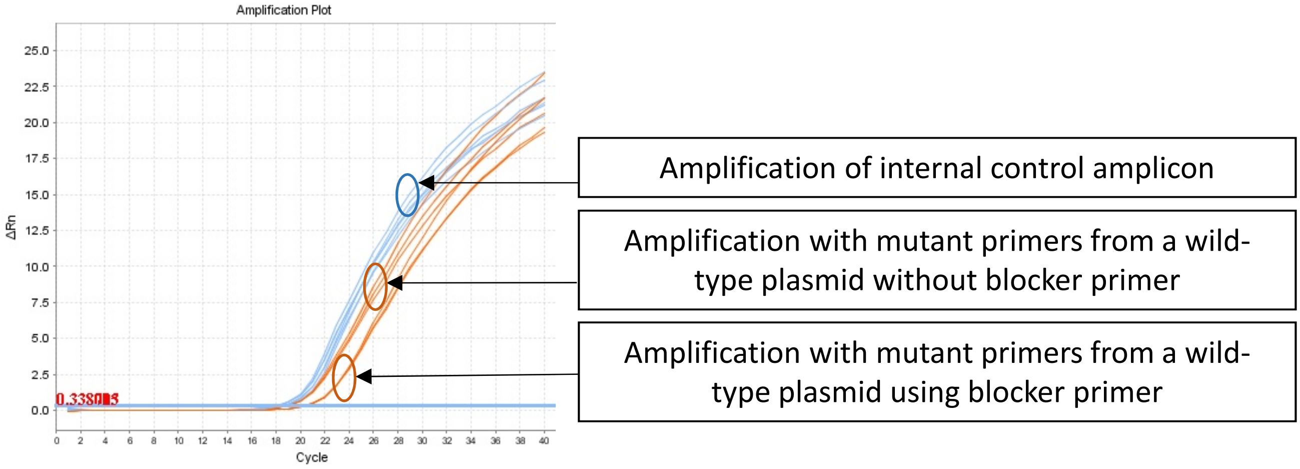

D

WT Plasmid	Ct MUT E545K	Ct internal control	ΔCt
Without blocker	22.42 ± 0.02	19.98± 0.30	2.44
With blocker	23.52 ± 0.22		3.54

ΔCt = Ct MUT E545K - Ct internal control

Supplementary Figure S2

A

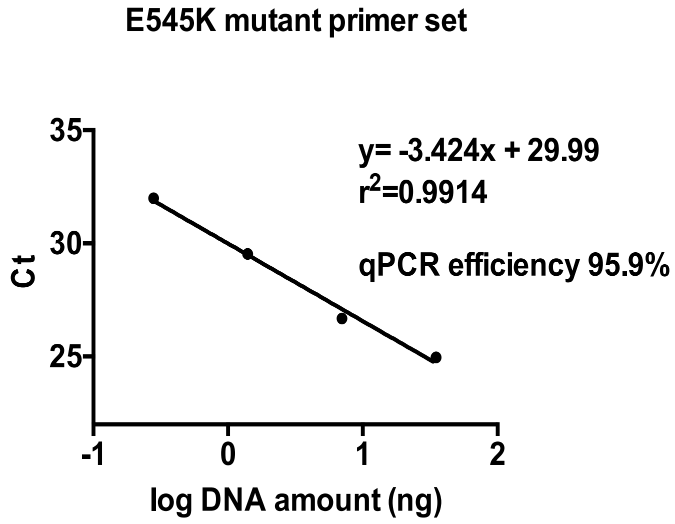

B

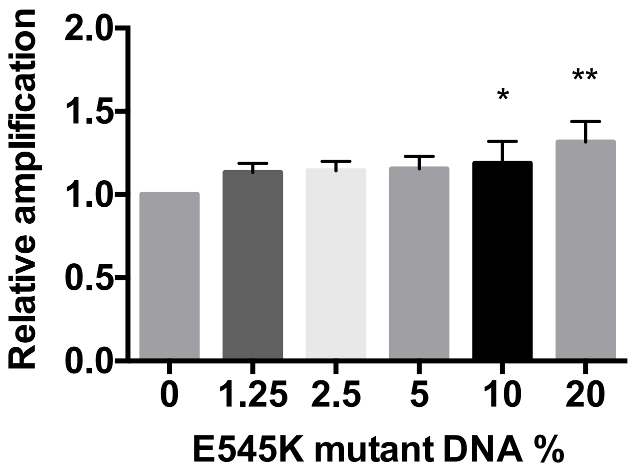

C

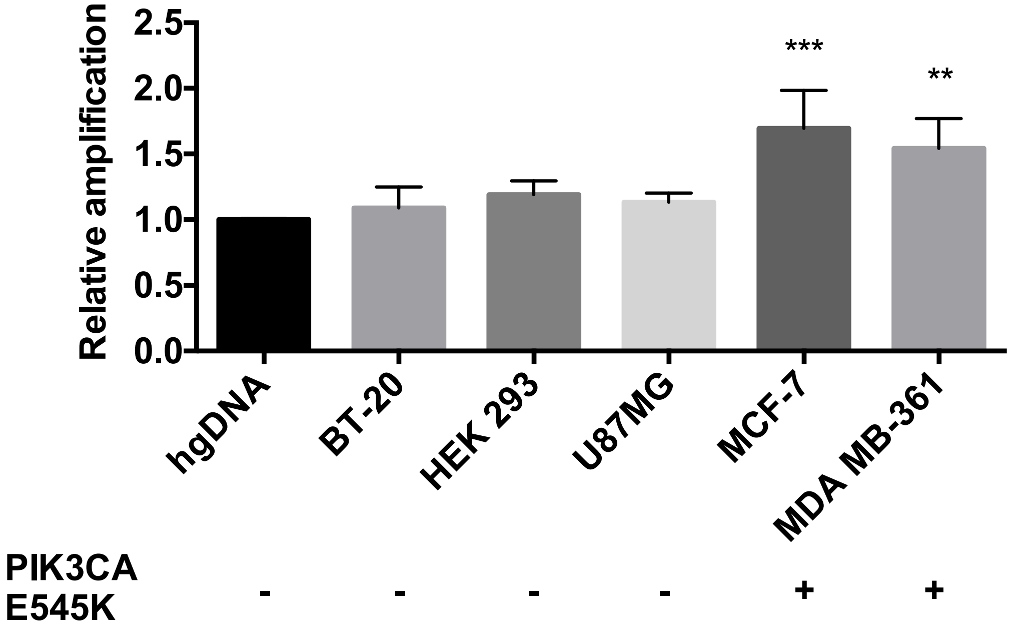

Supplementary table 1

Patient ID	Tumor characteristics	Grade	Tumor size (mm)	ER	PR	HER2
**B01**	Invasive carcinoma NST	2	13	7	8	-
**B02**	Invasive carcinoma NST	2	24	6	6	-
**B03**	Invasive carcinoma NST	3	13	8	7	-
**B04**	Invasive carcinoma NST	3	30	0	0	-
**B05**	Invasive carcinoma NST	2	15	8	8	-
**B06**	Invasive carcinoma NST	2	11	8	6	-
**B07**	Invasive carcinoma NST	3	23	8	8	-
**B08**	Invasive carcinoma NST	2	16	8	8	-
**B09**	Invasive carcinoma NST	3	23	7	4	+
**B10**	Invasive lobular carcinoma	2	22	8	5	-
**B12**	Invasive carcinoma NST	2	40	8	7	-
**B13**	Invasive carcinoma NST	3	23	7	8	-
**B14**	Invasive carcinoma NST	1	14	8	7	-
**B15**	Invasive carcinoma NST	2	18	8	7	-
**B16**	Invasive carcinoma NST	2	38	7	5	-
**B17**	Invasive carcinoma NST	3	19	8	0	-
**B18**	Invasive carcinoma NST	3	19	8	6	-
**B19**	Invasive carcinoma NST	2	11	8	8	-
**B20**	Invasive lobular carcinoma	2	22	8	7	-
**B21**	Invasive carcinoma NST	2	90	8	6	-
**B22**	Invasive carcinoma NST	2	19	8	7	-
**B23**	Invasive carcinoma NST	2	17	8	7	-

Abbreviations: ER (estrogen receptor), PR (progesterone receptor), HER2 (human epidermal growth factor receptor 2)

Supplementary table 2

Sample ID	Chromosome	Position	Ref-base	#Total Reads	#A	#C	#G	#T
**B02WBC**	chr3	178952085	A	4666	A:4657	C:1	G:6	T:2
**B03WBC**	chr3	178952085	A	3680	A:3674	C:0	G:6	T:0
**B07WBC**	chr3	178952085	A	2274	A:2268	C:0	G:6	T:0
**B09WBC**	chr3	178952085	A	3703	A:3692	C:0	G:10	T:1
**B10WBC**	chr3	178952085	A	4131	A:4114	C:1	G:10	T:6
**B12WBC**	chr3	178952085	A	3490	A:3477	C:0	G:9	T:4
**B13WBC**	chr3	178952085	A	5462	A:5441	C:0	G:18	T:3
**B14WBC**	chr3	178952085	A	2725	A:2717	C:2	G:2	T:4
**B15WBC**	chr3	178952085	A	2777	A:2767	C:0	G:6	T:4
**B16WBC**	chr3	178952085	A	2897	A:2880	C:3	G:6	T:8
**B17WBC**	chr3	178952085	A	4956	A:4928	C:1	G:19	T:8
**B20WBC**	chr3	178952085	A	3959	A:3946	C:0	G:11	T:2
**B21WBC**	chr3	178952085	A	4138	A:4128	C:0	G:10	T:0
**B22WBC**	chr3	178952085	A	3061	A:3059	C:0	G:2	T:0
**B23WBC**	chr3	178952085	A	3410	A:3397	C:0	G:10	T:1
**B02T**	chr3	178952085	A	3419	A:3400	C:0	G:14	T:5
**B03T**	chr3	178952085	A	4603	A:4583	C:2	G:8	T:10
**B07T**	chr3	178952085	A	4338	A:4325	C:3	G:8	T:2
**B09T**	chr3	178952085	A	4436	A:4415	C:1	G:18	T:2
**B10T**	chr3	178952085	A	5036	A:4997	C:0	G:32	T:5
**B12T**	chr3	178952085	A	3417	A:2522	C:0	G:892	T:3
**B13T**	chr3	178952085	A	4087	A:4059	C:2	G:16	T:10
**B14T**	chr3	178952085	A	3992	A:3977	C:3	G:12	T:0
**B15T**	chr3	178952085	A	3893	A:3869	C:3	G:14	T:7
**B16T**	chr3	178952085	A	3545	A:2759	C:0	G:784	T:2
**B17T**	chr3	178952085	A	2162	A:2146	C:0	G:14	T:2
**B20T**	chr3	178952085	A	5190	A:5182	C:1	G:5	T:2
**B21T**	chr3	178952085	A	4683	A:4674	C:0	G:7	T:2
**B22T**	chr3	178952085	A	5494	A:5469	C:0	G:19	T:6
**B23T**	chr3	178952085	A	4426	A:4207	C:3	G:213	T:3
